# *Ginkgo biloba* induced mood dysregulation: a case report

**DOI:** 10.1186/s12906-018-2081-4

**Published:** 2018-01-15

**Authors:** Seung Sun Rho, Young Sup Woo, Won-Myong Bahk

**Affiliations:** Department of Psychiatry, Yeouido St. Mary’s Hospital, College of Medicine, The Catholic University of Korea, 10, 63-ro, Seoul, 07345 Republic of Korea

**Keywords:** *Ginkgo biloba*, Mood dysregulation, Schizophrenia

## Abstract

**Background:**

Impairment of cognitive function as well as negative symptom is the major factor causing the decline of a patient’s functioning in chronic stages of schizophrenia. However, until now, there were no definite treatment options that could effectively reduce the impairment.

**Case presentation:**

We report a case of mood dysregulation associated with use of *Ginkgo biloba* in a patient with schizophrenia. After *Ginkgo biloba* was given, the patient experienced cluster symptoms of mood dysregulation including irritability, difficulty in controlling anger, agitation and restlessness. We estimated the possibility as “probable” according to Naranjo scale considering circumstantial evidence.

**Conclusions:**

This case suggests that *Ginkgo biloba* may have caused mood dysregulation in this patient. Although it is generally accepted as safe, more attention should be given to the adverse effect when treating with *Ginkgo biloba*.

## Background

Schizophrenia is a chronic and heterogeneous disease and the longitudinal course of the disease is gradually deteriorating [1]. Among various symptoms of schizophrenia, cognitive impairments which are often confused with negative symptoms and have been overlooked until recently, play a crucial role in the deterioration of patient functioning [2, 3]. Nowadays, awareness of its importance has led psychiatrists to consider treatment with a view to the cognitive aspects [4, 5]. For example, various compounds like acetyl-L-carnitine, omega-3 polyunsaturated fatty acids (PUFAs), N-acetylcysteine, positive allosteric modulators of metabotropic glutamate receptor 5 (mGluR5 PAMs), and vitamin E – which are known to improve mitochondrial functioning, have anti-oxidant properties, and stabilize neuronal membranes – have all been studied in various researches. However, the results are controversial [1, 6–9].

In this regard, there are a few reports that suggest *Ginkgo biloba* has anti-oxidant properties and could be used as an adjuvant for the treatment of schizophrenia. It is also considered fairly safe, as there are neither significant adverse effects, nor known drug interactions [10–14]. Nonetheless, *Ginkgo biloba* can potentially influence the serotonergic, adrenergic, and hypothalamic-pituitary-adrenal (H-P-A) axis system and thereby affect mood symptoms [15–23]. From this viewpoint, we would like to present a case of mood dysregulation associated with use of *Ginkgo biloba* in a patient with schizophrenia.

## Case presentation

Ms. J, a 50-year-old female, started her first psychiatric outpatient clinic when she was 29. She showed restricted affect at the time and was experiencing delusion of being controlled with auditory hallucination of a commanding nature, and was diagnosed as having schizophrenia. Risperidone was prescribed within the range of 2 to 6 mg with favorable response, and her symptoms gradually improved. However, after 2 years of remission periods, she decided on her own to discontinue taking the prescription, and this led to a recurrence of symptoms similar to those occurring at the time of her first episode. From then on, she has taken her prescription consistently and has been in stable condition. The patient has maintained remission with risperidone 2 mg monotherapy. In addition, she has never experienced any kind of mood episode.

In November 2016, the patient reported chronic cognitive discomfort, such as difficulty in concentration and short-term memory impairment. We first used acetyl-L-carnitine for 1 month and then it was switched to choline alfoscerate, both of which had no effect on her complaint. The prescription was thereafter switched to *Ginkgo biloba*, 80 mg twice a day. In the meantime, she was functioning well enough to get a part-time job. However, in January 2017, Ms. J reported symptoms such as irritability, difficulty in controlling anger, and agitation after taking *Ginkgo biloba* for 1 week. She said that these symptoms were the first she had experienced during her illness. She stopped taking *Ginkgo biloba* after these adverse events and the symptoms disappeared after about 2 or 3 days. During the same period, the patient continued taking risperidone 2 mg as usual and did not experience any of the previous thought problems or perceptual disturbances. Owing to these adverse reactions, she was instructed to discontinue the use of *Ginkgo biloba*. Nevertheless, 1 month later, in February 2017, the patient once again reported that she had tried *Ginkgo biloba* against our instructions because of the subjective cognitive discomfort which still remained. About 5 days after resuming *Ginkgo biloba* intake, she experienced the same symptoms of mood dysregulation such as irritability, difficulty in controlling anger, and agitation as earlier. She immediately stopped taking the drug and the symptoms disappeared within 2 days. She has remained stable after discontinuation of *Ginkgo biloba*. Her PANSS (Positive And Negative Syndrome Scale) and BPRS (Brief Psychiatric Rating Scale) scores were 40 and 4, respectively during her progress.

## Discussion

This is a report of a 50 year old female with chronic schizophrenia who experienced cognitive impairment presenting with mood dysregulation including symptoms of irritability, difficulty in controlling anger, anxiety, and agitation after using *Ginkgo biloba*. Whether patients with schizophrenia actually complain of cognitive decline or not, the reality is that many are likely to suffer from the impairment. This is challenging because there is no proven treatment option [1, 4, 5]. Nevertheless, based on the antioxidant effects of *Ginkgo biloba* [10, 11], there have been several clinical studies that have shown positive results when used as an adjuvant to antipsychotics in patients with chronic treatment resistant schizophrenia [12], especially in relieving negative symptoms [14].

In this case, we used acetyl-L-carnitine, choline alfoscerate, and *Ginkgo biloba* one by one sequentially. While the first two drugs did not have any effect, adverse mood symptoms developed with *Ginkgo biloba* use. To the best of our knowledge, there is only one similar case of hypomania in which a patient with a traumatic brain injury showed symptoms such as agitation, sleep disturbance, racing thoughts, and pressured speech after using *Ginkgo biloba* and St. John’s wort [24]. However, considering that St. John’s wort has proven antidepressant-like effects, it and not the *Ginkgo biloba*, could have been the cause of hypomanic symptoms [25]. Therefore, this may be the first case to show that *Ginkgo biloba* alone can have a causal relationship with mood symptoms but not hypomania.

On the other hand, there are some opposing clinical results in the literature. For instance, *Ginkgo biloba* was not shown to prevent depression in a placebo-controlled study by Lingaerde O et al. [26]. In other randomized controlled trials with dementia, although *Ginkgo biloba* was relatively effective in alleviating apathy/indifference and depression/dysphoria, it also seemed to reduce psychiatric symptoms such as irritability/lability and anxiety, which stand in contrast to our case [27, 28]. Therefore, the impact of *Ginkgo biloba* on mood dysregulation may be considered from the viewpoint that it has a low clinical evidence base.

The main pharmacological component of *Ginkgo biloba* is extract EGb (Extract *Ginkgo biloba*) 761. The exact mechanism of action is poorly understood, but it is basically known to have the following effects: vaso-regulatory activity resulting in increased blood flow to cerebral vessels; platelet activating factor antagonism; changes in neuronal metabolism and beneficial influence of neurotransmitter disturbance; and free-radical scavenging properties [10]. In addition, there is no significant difference in side effects compared to placebos. Only mild gastrointestinal discomfort, headache, and skin allergic reactions have been reported, and it also has no known special drug interactions [10, 29, 30].

Focusing more on the details, in studies on rats, *Ginkgo biloba* exerted an effect on neuronal membranes to restore 5-HT (5-Hydroxytryptamine) 1A receptors which decrease with aging, and it also protected against stress-induced desensitization of the receptors [15, 16]. Rat brain MAO (Monoamine Oxidase) A and B types were also reversibly inhibited by EGb 761, although this result was not reproduced in humans [17, 18]. Furthermore, *Ginkgo biloba* increased cerebral noradrenalin in another animal study [19] and it inhibited corticosteroid synthesis through a reduction in the number of adrenal peripheral benzodiazepine receptors, and interrupted the HPA axis by reducing CRH (Corticotropin-releasing hormone) expression [20–23]. These results indicate multiple possible mechanisms by which *Ginkgo biloba* could affect mood regulation.

There may be a question of reliability/validity on issues reported by schizophrenic patients [31]. It is known that scores of the self-rating scale are significantly different when repeatedly performed in those groups with cognitive impairment [32], although self-reporting is still regarded as useful in some ways [33]. Another limitation is that we did not perform any cognitive tests as we regarded her concentration and memory discomfort as being due to the cognitive symptoms of schizophrenia. However, considering the long remission period with low doses of risperidone, low PANSS and BPRS score, and fair socio-occupational functioning, her mood symptoms seem not to be associated with psychotic exacerbation, but they more likely suggest *Ginkgo biloba* relatedness The relationship between the use of *Ginkgo biloba* and mood events was assessed as “probable (7 points total)” when the Naranjo adverse drug reaction probability scale was applied [34]. In addition, the possibility of an adverse drug reaction was also “probable/likely” based on the WHO-UMC (World Health Organization-Uppsala Monitoring Center) causality categories [35].

## Conclusions

In conclusion, *Ginkgo biloba* may have affected the mood dysregulation of the patient when considering the evidence of prior studies, as well as the temporal relationship of the symptoms occurring after application of the medication, its interruption, and reapplication. The significance of this case suggests that *Ginkgo biloba*, which is generally accepted as safe, may nevertheless have adverse effects that merit attention. In this regard, further studies would be useful for supplementing our findings.

## Abbreviations

5-HT: 5-Hydroxytryptamine
BPRS: Brief Psychiatric Rating Scale
CRH: Corticotropin-releasing hormone
EGb: Extract *Ginkgo biloba*
H-P-A: Hypothalamic-pituitary-adrenal
MAO: Monoamine Oxidase
mGluR5 PAMs: Positive allosteric modulators of metabotropic glutamate receptor 5
PANSS: Positive And Negative Syndrome Scale
PUFAs: Polyunsaturated fatty acids
WHO-UMC: World Health Organization-Uppsala Monitoring Center
